# The diagnosis interval influences risk factors of mortality in patients with co-existent active tuberculosis and lung cancer: a retrospective study

**DOI:** 10.1186/s12890-023-02674-3

**Published:** 2023-10-10

**Authors:** Mengting Xiong, Shuanshuan Xie, Yukun Wang, Chenlei Cai, Wei Sha, Haiyan Cui, Jian Ni

**Affiliations:** 1 Clinic and Research Center of Tuberculosis, Department of oncology, Shanghai Pulmonary Hospital, School of Medicine, Tongji University, 507 Zheng Min Road, Shanghai, 200433 China; 2 Department of Respiratory Medicine, Shanghai Tenth People’s Hospital, Tongji University, Shanghai, 200072 China

**Keywords:** Tuberculosis, Lung cancer, The diagnosis interval, Survival

## Abstract

**Background:**

Previous studies reported that tuberculosis (TB) is associated with an increased risk of lung cancer or the survival and mortality of lung cancer. However, the impact of coexisting TB on the survival of lung cancer patients was controversial. We aimed to identify risk factors on the survival rate of patients with co-existent active TB and lung cancer.

**Methods:**

One hundred seventy-three patients diagnosed with active TB and lung cancer from January 2016 to August 2021 in Shanghai pulmonary hospital were selected and divided into two groups (≤ 6 months, > 6 months) according to the diagnosis interval between active TB and lung cancer (the order of diagnosis is not considered). The clinical characteristics and survival were analyzed. Univariate and multivariate logistic regression analyses were used to identify the risk factors for overall survival (OS).

**Results:**

One hundred seventy-three patients were diagnosed with lung cancer and active TB. The study population exhibited a median age of 64 years, with a majority of 81.5% being male, 58.0% of patients had a history of smoking. Among those involved, 93.6% had pulmonary TB, 91.9% were diagnosed with non-small cell lung cancer (NSCLC), 76.9% were Eastern Cooperative Oncology Group (ECOG) 0–2 and 12.7% were ECOG 3–4. We observed better survival in the > 6 months group compared with the ≤ 6 months group (hazard ratio [HR] 0.456, 95% confidence interval [CI]:0.234–0.889, *P* = 0.017). The 1-, 3-, and 5- year OS rates were 94.2%, 80.3%, and 77.6%, respectively, in the > 6 months group and 88.3%, 63.8%, and 58.5%, respectively, in the ≤ 6 months group. Surgery (HR 0.193, [95% CI, 0.038–0.097]; *P* = 0.046) and ECOG Performance Status (HR 12.866, [95% CI, 2.730–60.638]; *P* = 0.001) were independent prognostic factors in the > 6 months group.

**Conclusions:**

Patients diagnosed with lung cancer and active TB for more than half a year have a significantly better prognosis than those diagnosed within half a year. ECOG Performance Status and surgery might possibly affect the outcomes of patients with co-existent active TB and lung cancer.

## Introduction

Globally, TB ranks as the 13^th^ most prevalent cause of mortality and the second most significant infectious agent, resulting in 10 million newly reported cases and a total of 1.5 million fatalities in the year 2020 [1]. China possesses one of the most significant TB burdens, ranking third and accounting for 8.4% of the total global cases [2]. Lung cancer is the second most commonly diagnosed cancer [3] and the leading cause of cancer death, representing approximately 1 in 10 (11.4%) cancers diagnosed and 1 in 5 (18.0%) deaths [4]. In China, it is expected that there will be approximately 870,982 people newly diagnosed with lung cancer, and 766,898 people dying from lung cancer in 2022 [5].

Epidemiological studies have revealed that TB is associated with an increased risk of lung cancer [6, 7], or the survival and mortality of lung cancer [8–10], especially adenocarcinoma [11]. Cabrera-Sanchez J et al. have demonstrated that patients diagnosed with TB are at an elevated risk of developing lung cancer [6, 12]. Conversely, patients with cancer exhibit a higher incidence of TB [13]. In a retrospective cohort analysis, the adjusted hazard ratio (aHR) for TB in lung cancer patients was 3.32 [14]. However, the impact of coexisting TB on the survival of lung cancer patients was controversial. TB was independently associated with subsequent mortality due to lung cancer (adjusted HR = 2.01, 95%CI [1.40–2.90], *P* < 0.001) [15] in a cross-matched cohort. A Korean retrospective study reported that lung cancer with TB was associated with lower mortality (HR = 0.35, 95% CI [0.21–0.60]) [8]. Zhi-Hong Jian et al. reported that coexisting pulmonary diseases are at an elevated risk of mortality among male patients with lung adenocarcinoma [11].

Previous studies have investigated numerous risk factors associated with the co-existence of TB and lung cancer, including smoking [16, 17], age [18], gender [19], inflammatory cytokines [20], C-reactive protein [21]. The objective of this study was to evaluate the impact of these factors on the survival rate of patients with co-existent TB and lung cancer according to the diagnosis interval.

## Methods

The present study was performed in Shanghai pulmonary hospital, the standard authority for the diagnosis and treatment of TB and lung cancer in China. The study was approved by the ethics committee of Shanghai pulmonary hospital (Identifier: K18-145).

### Study design and population

We conducted a single-center, retrospective analysis of patients with coexisting lung cancer and TB. Patients diagnosed with lung cancer and active TB were enrolled in Shanghai pulmonary hospital from January 2016 to August 2021. Clinical signs, demographic, biological and imaging data were retrieved from the patients’ electronic hospital records. Patient with other tumors or suspected TB or non-tuberculosis mycobacteria (NTM) were excluded from this study. Based on the diagnosis interval, patients were divided into two groups: ≤ 6 months, > 6 months (the order of diagnosis is not considered). Diagnosis of active TB was confirmed by bacteriologic, pathologic, radiographic, and clinical evidence. Baseline patient characteristics were collected, including age, genders, smoking status, stage, comorbidities, cancer type, tumor location, CT image, treatments, the laboratory findings and survival. Clinical staging was performed using the 7th edition of the TNM staging system, which was authorized by the American Joint Committee on Cancer [22].

### Outcome

The primary outcome was death from any cause. Follow-up time was calculated from the date of lung cancer diagnosis till date of death or end of the follow-up period on March 31, 2022.

### Statistical analysis

Analyses were conducted in SPSS (26.0, SPSS, Chicago, USA) and R (V.3.6.0; The R Project for Statistical Computing).

Baseline characteristics were described with frequencies and percentages for categorical variables and means and standard deviations (SDs) for continuous variables, whereas the median and interquartile range (IQR: 25th–75th) were used for non-normally distributed data. Analysis of the differences between the diagnosis interval ≤ or > 6 months group was performed using the Student’s t-test for continuous variables and the χ^2^ test or Fisher’s exact test for categorical variables. Kaplan–Meier (KM) analysis and the log rank test were applied to compare OS between two groups. A Cox proportional hazards model was used to test for significant factors on survival when the variables were significantly different at *P* ≤ 0.05 in the log-rank test with a univariate or multivariate analysis in each group.

## Result

### Patient characteristics

**Fig. 1:**
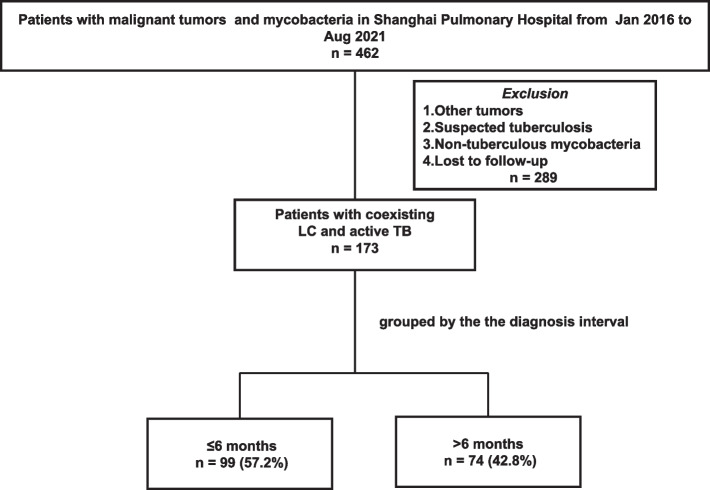
Flowchart of the study population. Abbreviation: TB, tuberculosis

One hundred seventy-three patients diagnosed with lung cancer and active TB from Jan 2016 to August 2021 were divided into two groups based on the diagnosis interval, ≤ 6 months (*n* = 99), > 6 months (*n* = 74) (Fig. 1). Population characteristics are outlined in Table 1. The study population exhibited a median age of 64 years, with a majority of 81.5% being male, 58.0% of patients had a history of smoking. Among those involved, 93.6% had pulmonary TB, 91.9% were diagnosed with non-small cell lung cancer (NSCLC), 76.9% were ECOG 0–2 and 12.7% were ECOG 3–4. Patients with early and late stage tumors accounted for the majority (stage I 28.9%; stage IV 33.5%). Patients in the two groups had comparable characteristics, including age, sex, smoking status, stage, comorbidities, tumor location, CT image. The laboratory findings, including C-reactive protein (CRP), erythrocyte sedimentation rate (ESR), inflammatory cytokines, CD4/CD8 T cell ratio were nearly similar in both groups. The two groups of anti-TB treatment accounted for a similar proportion. However, squamous cell carcinoma was found more often in the ≤ 6 months group. Compared with ≤ 6 months group, more patients in the > 6 months group were treated with surgery and chemotherapy. ECOG 0–2, ECOG 3–4 were 49.6%, 15.2%, respectively, in the ≤ 6 months group and 90.5%, 9.5%, respectively, in the > 6 months group (*P* = 0.000). The mean follow-up period was 14 months (range, 11–19 months) in the ≤ 6 months group and 29 months (range, 8.5–62 months) in the > 6 months group (*P* = 0.000).

**Table 1:** Baseline characteristics (*N* = 173)

		**the diagnosis interval**		
**Characteristic**	**Total(*N* = 173)**	** ≤ 6 months(*N* = 99)**	** > 6 months(*N* = 74)**	***P Value***
Status, n (%)				
Living	97(56.1)	52(52.5)	45(60.8)	0.568
Died	35(20.2)	22(22.2)	13(17.6)	
Unknown	41(23.7)	25(25.3)	16(21.6)	
Gender, n (%)				
Male	141(81.5)	82(82.8)	59(79.7)	0.693
Female	32(18.5)	17(17.2)	15(20.3)	
Age, y				
Median (IQR), y	64(58.5,69.5)	65 (59,69)	63(58,70)	0.528
< 65, n (%)	89(51.4)	46(46.5)	43(58.1)	0.130
≥ 65, n (%)	84(48.6)	53(53.5)	31(41.9)	
Smoking, n (%)				
Yes	97(56.1)	54(54.5)	43(58.1)	0.726
No	49(28.3)	29(29.3)	20(27.0)	
Unknown	27(15.6)	16(16.2)	11(14.9)	
ECOG Performance				0.000
Status, n (%)				
0–2	133(76.9)	66(49.6)	67(90.5)	
3–4	22(12.7)	15(15.2)	7(9.5)	
Unknow	18(10.4)	18(18.2)	0(0.0)	
Comorbidities, n (%)				
DM	27(15.6)	14(14.1)	13(17.6)	0.673
HTN	25(14.5)	14(14.1)	11(14.9)	1.000
COPD/Emphysema	48(27.7)	32(32.3)	16(21.6)	0.123
CHD/Arrthythmia	10(5.8)	5(5.1)	5(6.8)	0.746
Bronchiectasis	11(6.4)	6(6.1)	5(6.8)	1.000
Liver disease	6(3.5)	3(3.0)	3(4.1)	1.000
Stoke	7(4)	4(4.0)	3(4.1)	1.000
Immunodeficiency	3(1.7)	3(3.0)	0(0.0)	0.261
Other cancers	5(2.9)	3(3.0)	2(2.7)	1.000
Cancer type, n (%)				
SCLC	12(6.9)	8(8.1)	4(5.4)	0.017
NSCLC	159(91.9)	91(91.9)	68(91.9)	
Adenocarcinoma	93(53.8)	46(46.5)	47(63.5)	
Squamous cell carcinoma	55(31.8)	40(40.4)	15(20.3)	
Undifferentiated	11(6.4)	5(5.1)	11(14.9)	
Unknown	2(1.2)	0(0.0)	2(2.7)	
Stage, n (%)				
I	50(28.9)	27(27.3)	23(31.1)	0.332
II	8(4.6)	4(4.0)	4(5.4)	
III	33(19.1)	20(20.2)	13(17.6)	
IV	58(33.5)	38(38.4)	20(27.0)	
Unknown	24(13.9)	10(10.1)	14(18.9)	
Tumor location, n (%)				
Bilateral	13(7.5)	7(7.1)	6(8.1)	0.103
Right	86(49.7)	56(56.6)	30(40.5)	
Left	74(42.8)	36(36.4)	38(51.4)	
System involved, n (%)				
Pulmonary and extrapulmonary	6(3.5)	0(0.0)	6(8.1)	0.008
Pulmonary only	162(93.6)	96(97.0)	66(89.2)	
Extrapulmonary only	5(2.9)	3(3.0)	2(2.7)	
Cavity, n (%)	47(27.2)	27(27.3)	20(27.0)	1.000
Retreatment TB, n (%)	17(9.8)	8(8.1)	9(12.2)	0.442
Treatment, n (%)				
Surgery	68(39.3)	24(24.2)	44(59.5)	0.000
Chemotherapy	50(28.9)	22(22.2)	28(37.8)	0.011
Radiation	20(11.6)	8(8.1)	12(59.5)	0.118
targeted therapy	15(8.7)	7(7.1)	8(10.8)	0.559
Anti-PD-(L)1 therapy	7 (4)	4(4.0)	3(4.1)	0.878
Anti-TB treatment	168 (97.1)	96 (97.0)	72 (97.3)	0.396
CRP(mg/L),n (%)				
< 10	49(28.3)	29(29.3)	20(27.0)	0.641
10–50	29(16.8)	14(14.1)	15(20.3)	
> 50	26(15.0)	14(14.1)	12(16.2)	
Unknown	69(39.9)	42(42.4)	27(36.5)	
IL-1β, n (%)				
normal	48(27.7)	25(25.3)	23(31.1)	0.692
Higher than normal	14(8.1)	8(8.1)	6(8.1)	
unknown	111(64.2)	66(66.7)	45(60.8)	
IL-2R, n (%)				
Normal	30(17.3)	18(18.2)	12(16.2)	0.437
Higher than normal	32(18.5)	15(15.2)	17(23.0)	
unknown	111(64.2)	66(66.7)	45(60.8)	
IL-6, n (%)				
normal	16(9.2)	9(9.1)	7(9.5)	0.762
Higher than normal	46(26.6)	24(24.2)	22(29.7)	
unknown	111(64.2)	66(66.7)	45(60.8)	
TNF-α, n (%)				
Normal	14(8.1)	6(6.1)	8(10.8)	0.531
Higher than normal	42(24.3)	25(25.3)	17(23.0)	
unknown	117(67.6)	68(68.7)	49(66.2)	
IGRA, n (%)				
negative	17(9.8)	11(11.1)	6(8.1)	0.766
positive	127(73.4)	71(71.7)	56(75.7)	
unknown	29(16.8)	17(17.2)	12(16.2)	
SAA(IQR), mg/L	11(6.3,86.3)	7.3(3.2,20.9)	14.2(8.5,84.1)	0.364
ESR(IQR), mm/h	50(25,81.5)	36(24,68)	54(15,71)	0.304
Lymphocyte count (SD) *10 ~ 9	1.3(0.6)	1.3(0.5)	1.2(0.6)	0.714
CD4 + /CD8 + T-cell ratio (IQR)*10 ~ 9	2(1.3,2.9)	2.2(1.3,3.2)	2(1.5,2.4)	0.382
Survival time (IQR), M	21(9,49.5)	14(11,19)	29(8.5,62)	0.000

*Abbreviations*: *ECOG* Eastern Cooperative Oncology Group, *DM* diabetes mellitus, *HTN* hypertension, *COPD* chronic obstructive pulmonary disease, *CHD* coronary heart disease, *SCLC* small-cell lung cancer, *NSCLC* non-small cell lung cancer, *TB* tuberculosis, *CRP* C-reactive protein, *IL* interleukin, *TNF* tumor necrosis factor, *IGRA* interferon gamma release assay, *SAA* Serum amyloid a, *IQR* inter-quartile range, *SD* standard deviation, *ESR* erythrocyte sedimentation rate

### Survival

**Fig. 2:**
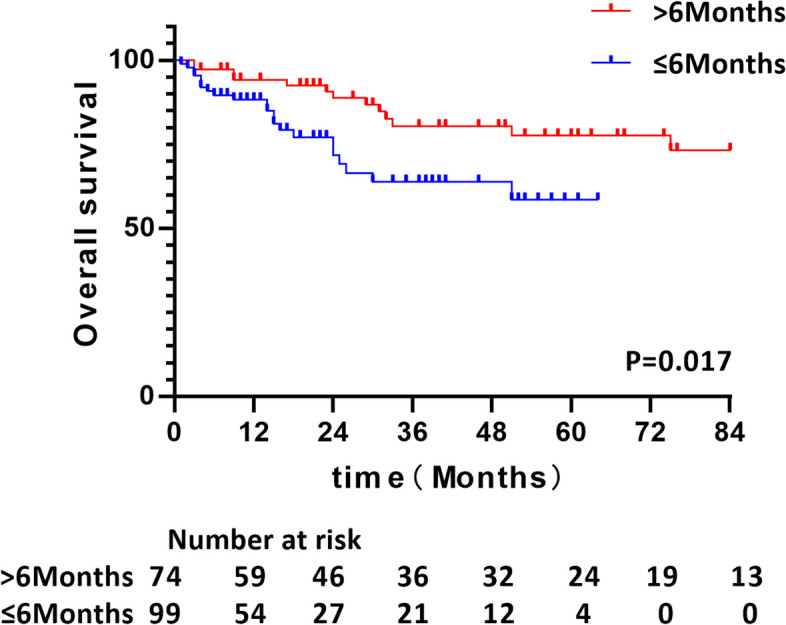
Kaplan–Meier curves of overall survival according to the diagnosis interval between active TB and lung cancer. Numbers of patients at risk are indicated for the ≤ 6 months and > 6 months groups. Abbreviation: TB, tuberculosis

**Fig. 3:**
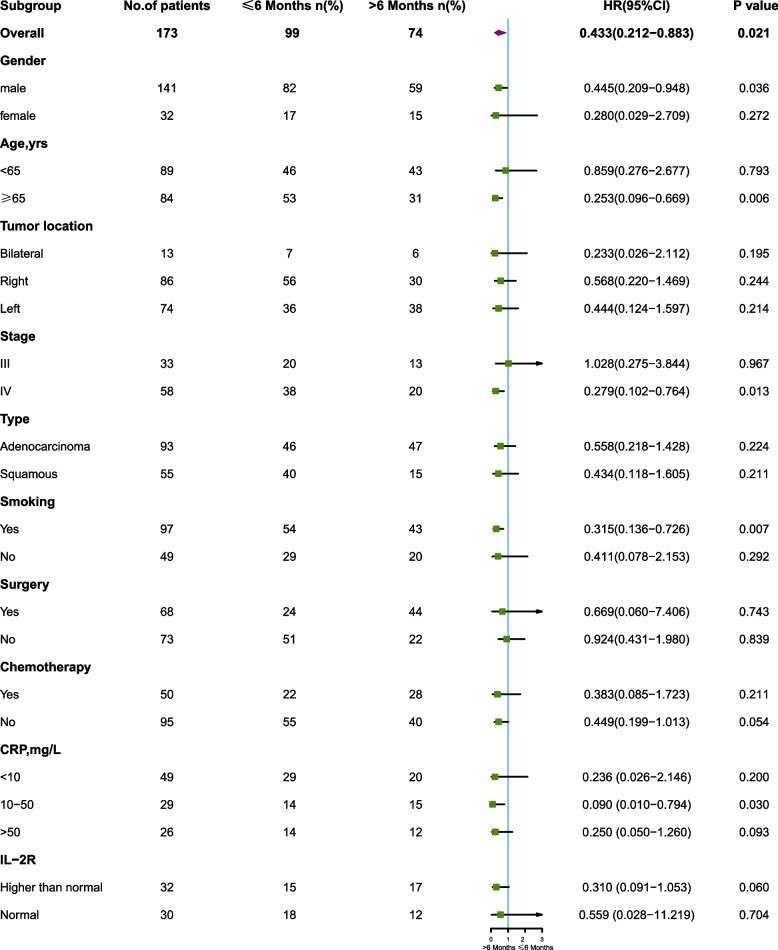
Forest plot of HRs of factors that can influence OS in subgroup analysis. Abbreviation: HR, hazard ratio; CI, confidence interval; OS, overall survival; CRP, C-reactive protein; IL, interleukin

Patients in the > 6 months group consistently were found to achieve better survival than that in the ≤ 6 months group (HR = 0.456, 95% CI [ 0.234–0.889], *P* = 0.017). The 1-, 3-, and 5- year OS rates were 94.2%, 80.3%, and 77.6%, respectively, in the > 6 months group and 88.3%, 63.8%, and 58.5%, respectively, in the ≤ 6 months group (Fig. 2). Separate analyses in subsets of patients according to gender, age, tumor location, stage, and type, smoking, treatments, the laboratory findings revealed the same pattern, with few exceptions. We found that patients older than 65 years old (HR 0.253, [95% CI, 0.096–0.669]; *P* = 0.006), male (HR 0.445, [95% CI, 0.209–0.948]; *P* = 0.036), stage IV (HR 0.279, [95% CI, 0.102–0.764]; *P* = 0.013), history of smoking (HR 0.315, [95% CI, 0.136–0.726]; *P* = 0.007), and those with the level of CRP range from 10 mg/L to 50 mg/L (HR 0.090, [95% CI, 0.010–0.794]; *P* = 0.03) were all significantly more likely to have worse cumulative OS rate, in the ≤ 6 months group compared with the > 6 months group (Fig. 3).

### Univariate and multivariate analysis

Significant predictors were further assessed with a multivariable Cox proportional hazards model after evaluation by the univariate analysis when* P* < 0.05 in both groups. In the ≤ 6 months group, all the variables, age (HR 0.294, [95% CI, 0.114–0.760]; *P* = 0.011), left lung cancer (HR 0.271, [95% CI, 0.075–0.973]; *P* = 0.045), smoking (HR 3.519, [95% CI, 1.267–9.774]; *P* = 0.016), surgery (HR 0.077, [95% CI, 0.010–0.577]; *P* = 0.013), ECOG Performance Status (HR 16.329, [95% CI, 6.598–40.415]; *P* < 0.001), CRP 10–50 mg/L (HR 10.397, [95% CI, 1.288–83.951]; *P* = 0.028), IL-2R (HR 9.376, [95% CI, 1.122–78.323]; *P* = 0.028) were significant risk factors affecting OS in the univariate analysis. After multivariate Cox proportional hazards regression analyses, no variable was identified as an independent prognostic factor (Table 2).

**Table 2:** Cox survival analysis in the ≤ 6 months group

Variable	Univariate Analysis		Multivariate Analysis	
	HR (95%CI)	*P* Value	HR (95%CI)	*P* Value
Gender(male)	2.550(0.745–8.730)	0.136		
Age, y (≥ 65)	0.294(0.114–0.760)	0.011	0.237(0.016–3.591)	0.299
Tumor location		0.125		
Bilateral	Reference			
Right	0.386(0.123–1.207)	0.102		
Left	0.271(0.075–0.973)	0.045		
Cancer type		0.627		
SCLC	Reference			
Adenocarcinoma	1.037(0.132–8.161)	0.973		
Squamous-cell carcinoma	1.818(0.232–14.255)	0.569		
Stage		0.320		
I	Reference			
II	N/A	0.999		
III	N/A	0.915		
IV	N/A	0.906		
Unknown	N/A	0.910		
Smoking	3.519(1.267–9.774)	0.016	0.462(0.038–5.568)	0.544
System involved		0.870		
Pulmonary only	Reference			
Extrapulmonary only	1.182(0.158–8.821)			
Tuberculosis retreatment		0.977		
No	Reference			
Yes	1.031(0.135–7.843)			
Anti-PD-(L)1 therapy		0.579		
No	Reference			
Yes	0.037(N/A)			
Surgery	0.077(0.010–0.577)	0.013	0.000(N/A)	0.946
ECOG Performance Status		< 0.001		0.081
0–2	Reference		Reference	
3–4	16.329(6.598–40.415)		12.619(0.732–217.420)	
Chemotherapy		0.268		
No	Reference			
Yes	0.542(0.183–1.603)			
Targeted therapy		0.313		
No	Reference			
Yes	0.355(0.048–2.649)			
Radiotherapy		0.739		
No	Reference			
Yes	1.231(0.362–3.190)			
CRP(mg/L),n (%)		0.083		
< 10	Reference			
10–50	10.397(1.288–83.951)	0.028		
> 50	2.598(0.463–14.587)	0.278		
IL-1β		0.969		
Normal	Reference			
Higher than normal	0.968(0.187–5.017)			
IL-2R		0.039		0.947
Normal	Reference		Reference	
Higher than normal	9.376(1.122–78.323)		1293597.415(N/A)	
IL-6		0.179		
Normal	Reference			
Higher than normal	4.305(0.512–36.198)			
TNF-α		0.564		
Normal	Reference			
Higher than normal	25.642(N/A)			
IGRA		0.917		
Negative	Reference			
Positive	0.936(0.273–3.211)			
Cavity		0.100		
No	Reference			
Yes	2.096(0.867–5.065)			

*Abbreviations***:** *SCLC* small-cell lung cancer, *ECOG* Eastern Cooperative Oncology Group, *CRP* C-reactive protein, *IL* interleukin, *TNF* tumor necrosis factor, *IGRA* interferon gamma release assay, *HR* hazard ratio, *CI* confidence interval

A *p*-value of less than 0.05 represents a significant statistical difference

In the > 6 months group, stage III (HR 15.192, [95% CI, 1.675–137.826]; *P* = 0.016), stage IV (HR 12.715, [95% CI, 1.556–103.866]; *P* = 0.018), surgery (HR 0.079, [95% CI, 0.021–0.296]; *P* = 0.000), ECOG Performance Status (HR 39.118, [95% CI, 9.852–155.313]; *P* < 0.001), CRP > 50 mg/L (HR 11.485, [95% CI, 1.165–113.215]; *P* = 0.037) were significant predictors in the univariate analysis. Multivariate Cox proportional hazards regression analyses showed that surgery (HR 0.193, [95% CI, 0.038–0.097]; *P* = 0.046), ECOG Performance Status (HR 12.866, [95% CI, 2.730–60.638]; *P* = 0.001) were independent prognostic factors (Table 3).

**Table 3:** Cox survival analysis in the > 6 months group

Variable	Univariate Analysis		Multivariate Analysis	
	HR (95%CI)	*P* Value	HR (95%CI)	*P* Value
Gender(male)	5.540(0.712–43.083)	0.102		
Age, y (≥ 65)	1.464(0.492–4.357)	0.494		
Tumor location		0.701		
Bilateral	Reference			
Right	1.256(0.154–10.279)	0.832		
Left	0.765(0.075–0.973)	0.807		
Cancer type		0.987		
SCLC	Reference			
Adenocarcinoma	N/A	0.947		
Squamous-cell carcinoma	N/A	0.945		
Stage		0.067		
I	Reference			
II	N/A	0.987		
III	15.192(1.675–137.826)	0.016		
IV	12.715(1.556–103.866)	0.018		
Unknown	2.047(0.128–32.788)	0.613		
Smoking	3.525(0.768–16.179)	0.105		
System involved		0.636		
Pulmonary only	Reference			
Extrapulmonary only	N/A	0.669		
Pulmonary and extrapulmonary	N/A	0.395		
Tuberculosis retreatment		0.509		
No	Reference			
Yes	0.502(0.065–3.872)			
Anti-PD-(L)1 therapy		0.731		
No	Reference			
Yes	1.430(0.186–11.010)			
Surgery	0.079(0.021–0.296)	< 0.001	0.193(0.038–0.970)	0.046
ECOG Performance Status		< 0.001	12.866(2.730–60.638)	0.001
0–2	Reference			
3–5	39.118(9.852–155.313)			
Chemotherapy		0.685		
No	Reference			
Yes	0.783(0.241–2.546)			
Targeted therapy		0.859		
No	Reference			
Yes	0.872(0.192–3.968)			
Radiotherapy		0.444		
No	Reference			
Yes	0.450(0.058–3.470)			
CRP(mg/L),n (%)		0.112		
< 10	Reference			
10–50	6.762(0.598–76.462)	0.122		
> 50	11.485(1.165–113.215)	0.037		
IL-1β		0.506		
Normal	Reference			
Higher than normal	0.488(0.059–4.060)			
IL-2R		0.185		
Normal	Reference			
Higher than normal	4.202(0.503–35.074)			
IL-6		0.312		0.967
Normal	Reference		Reference	
Higher than normal	35.307(N/A)		N/A	
TNF-α		0.854		
Normal	Reference			
Higher than normal	1.230(0.135–11.173)			
IGRA		0.813		
Negative	Reference			
Positive	1.286(0.159–10.383)			
Cavity		0.517		
No	Reference			
Yes	1.927(0.629–5.903)	0.251		

*Abbreviations*: *SCLC* small-cell lung cancer, *ECOG* Eastern Cooperative Oncology Group, *CRP* C-reactive protein, *IL* interleukin, *TNF* tumor necrosis factor, *IGRA* interferon gamma release assay, *HR* hazard ratio, *CI* confidence interval. A *p*-value of less than 0.05 represents a significant statistical difference

## Discussion

In this retrospective study, patients in the > 6 months group were consistently found to have a better prognosis than patients in the ≤ 6 months group. This survival advantage was independent of differences in baseline characteristics such as gender, age, stage, and type, smoking, treatments, and the laboratory findings.

Sex-related differences exist in many lung diseases [23] including TB and lung cancer. Previous studies revealed females with active pulmonary TB had a higher risk of dying from lung cancer than males [16, 19]. In China, the mortality ratio was 1.72 for males and 2.79 for females [24]. However, a study from Taiwan, which showed that coexisting pulmonary disease may exert direct effects and increase risk of mortality in men, but not in women [11]. In our study, overall female had a better prognosis than men in overall. Meanwhile, we found that the diagnosis interval can affect prognosis in men, but not in women.

Several studies have investigated the incidence of lung cancer and TB, with a trend towards younger people at the age of high incidence. A South Korean cohort study has revealed that the risks for lung cancer were HR 9.85, 7.1, 3.32, and 2.57 in patients with TB aged 50–59, 60–69, and ≥ 70 years, respectively, compared to patients < 50 years of age [18]. An et al. have found that the mean age of patients with co-existence of TB and lung cancer was 69 years and the risk of lung cancer subsequent to pulmonary TB was significantly higher both for patients younger than 60 years and for those older than 60 years [25], which is consisted with our findings. We found that the diagnosis interval had a greater impact on the prognosis in the elderly, especially those whose diagnosis interval were less than half a year.

Smoking is the most important environmental risk factor for both lung cancer and TB [26]. However, the association between smoking and the development of lung cancer and TB remains uncertain [19, 27]. Liang et al. and Hwang et al**.** [27] suggested that smoking was not only an influential factor in the development increased risk of of lung cancer in patients with preexisting TB [28]. In a retrospective cohort study conducted in Xuanwei [29], the mortality was similar among men after adjustment for smoking status (HRs 9.7 and 4.3 in the 0–4.9 years and 5 + years after tuberculosis, respectively) and among women after adjustment for smoky coal use (HRs 7.5 and 2.5, respectively). Among ever-users of smoky coal (*N* = 2430 lung cancer deaths), TB was associated with higher risk of mortality from lung cancer, specifically within the first 0–4.9 years after TB diagnosis (HR 7.5, 95% CI 4.9–11). Additionally in the 5 + years following TB diagnosis, the HR was 2.5 with a 95% CI of 1.2–5.0. In our study, smoking increased the mortality in two groups and the > 6 months group had a better prognosis than the ≤ 6 months group in ever-smokers.

Mycobacterium TB can induce the release of inflammatory mediators, e.g., tumor necrosis factor (TNF)-α and interleukin (IL)-1, IL-2, and IL-12, which can be viewed as cancer promotors [30]. Several studies identified that high CRP levels were risk factors for the development of lung cancer and elevated serum CRP levels will increase the incidence of lung cancer in male TB patients [21]. We found that the number of patients with normal CRP and IL-2 levels were comparable to that of abnormal patients, and that mortality was higher in the < 6 months group among patients with CRP levels of 10 to 50 mg/L.

Lee et al. [8] and Kim et al. [31] reported that much higher proportions of lung cancer coexisting with pulmonary TB were at an advanced stage (T3-4), which was generally consistent with our findings, but we also found that the proportion of phase I and phase IV was comparable and the diagnosis interval can affect the prognosis of advanced patients. TB was found to be significantly associated with adenocarcinoma, but not with squamous or small-cell lung cancer (SCLC) [19]. In our study, adenocarcinoma accounted for the majority, especially in the > 6 months group, however, the type of pathology had no effect on patient prognosis in both groups.

The treatment of patients with lung cancer and TB is still not conclusive. Previous studies reported that lung cancer patients with co-existent granulomatous inflammation who had undergone surgical resection suggest a relatively good clinical outcome even without anti-TB treatment [32]. As for chemotherapy, there was no significant difference in treatment regimen, response rate, median survival time [33] in lung cancer and lung cancer patients with co-existent TB. We found surgery was a positive prognostic indicator in both groups, and an independent factor in the > 6 months group, while chemotherapy had no effect. Short-term tuberculosis lesions and lung cancer lesions are difficult to distinguish, which may result in delayed staging of lung cancer and the inability to perform timely surgery, leading to poor survival and prognosis for patients [34]. Therefore, when diagnosing lung cancer in the presence of active TB, it is imperative to conduct a more precise evaluation of lung cancer staging. This can be achieved through various methods such as Positron Emission Tomography-Computed Tomography, lung puncture, bronchoscopy, etc., rather than solely relying on Chest Computed Tomography..

Few studies analyzed the relationship between ECOG and the prognosis of patients with coexisting lung cancer and active TB. Only a recent study found that lung cancer, presence of metastasis and ECOG ≥ 3 were associated with death from TB [35], which is consisted with our results. In our study, ECOG 0–2 accounted for the majority, especially in the > 6 months group. Meanwhile, we found ECOG 3–4 was an independent risk factor in the > 6 months group. Simultaneous administration of anti-tumor and anti-tuberculosis treatment within a short period of time may exacerbate the patient’s ECOG performance status, preventing them from proceeding to the next step of treatment and resulting in poorer survival and prognosis. Therefore, it is advisable to select treatments with minimal impact on the patient's ECOG score.

Our study have a number of strengths. This study is one of the few retrospective studies that analyses the relationship between the diagnosis interval and the prognosis of patients with coexisting lung cancer and active TB [8, 34]. Previous studies have focused on the effects of pulmonary TB on the development and treatment of lung cancer [6, 20, 33, 34]. Secondly, we assessed various risk factors as possible, along with other potential factors, for better control to measure confounding and report unbiased results. Lastly, this study enrolled a relatively large number of patients with coexisting lung cancer and active TB.

Our study includes several limitations that may influence its generalizability. Firstly, it was performed retrospectively at a single center, and therefore the results may not reflect the general population in China. Secondly, we did not further group the order of diagnosis of lung cancer and pulmonary TB, which can analyze the interaction between TB and lung cancer. The possibility of sampling bias in the diagnosis of active TB could not be excluded. Patients with lung cancer usually received more medical attention. In addition, our study follow-up period was relatively shorter.

## Conclusion

Patients diagnosed with lung cancer and active TB for more than half a year have a significantly better prognosis than those diagnosed within half a year. ECOG Performance Status and surgery might possibly affect outcomes of patients with co-existent active TB and lung cancer.

## Data Availability

All data generated or analyzed during this study are included in this published article. The data that support the findings of this study are available from the corresponding author upon reasonable request.

## Abbreviations

TB: Tuberculosis
NTM: Non-tuberculosis mycobacteria
OS: Overall survival
DM: Diabetes mellitus
HTN: Hypertension
COPD: Chronic obstructive pulmonary disease
CHD: Coronary heart disease
ECOG: Eastern Cooperative Oncology Group
SCLC: Small-cell lung cancer
NSCLC: Non-small cell lung cancer
CRP: C-reactive protein
IL: Interleukin
TNF: Tumor necrosis factor
IGRA: Interferon gamma release assay
SAA: Serum amyloid a
IQR: Inter-quartile range
SD: Standard deviation
ESR: Erythrocyte sedimentation rate
HR: Hazard ratio
CI: Confidence interval
